# Bexarotene targets autophagy and is protective against thromboembolic stroke in aged mice with tauopathy

**DOI:** 10.1038/srep33176

**Published:** 2016-09-14

**Authors:** Mikko T. Huuskonen, Sanna Loppi, Hiramani Dhungana, Velta Keksa-Goldsteine, Sighild Lemarchant, Paula Korhonen, Sara Wojciechowski, Eveliina Pollari, Piia Valonen, Juho Koponen, Akihiko Takashima, Gary Landreth, Gundars Goldsteins, Tarja Malm, Jari Koistinaho, Katja M. Kanninen

**Affiliations:** 1 Department of Neurobiology, A.I. Virtanen Institute for Molecular Sciences, University of Eastern Finland, Kuopio, Finland; 2 Department of Life Science, Gakushuin University, Toshiba-ku, Tokyo, Japan; 3 Department of Neurosciences, Case Western Reserve University School of Medicine, Cleveland, Ohio, USA

## Abstract

Stroke is a highly debilitating, often fatal disorder for which current therapies are suitable for only a minor fraction of patients. Discovery of novel, effective therapies is hampered by the fact that advanced age, primary age-related tauopathy or comorbidities typical to several types of dementing diseases are usually not taken into account in preclinical studies, which predominantly use young, healthy rodents. Here we investigated for the first time the neuroprotective potential of bexarotene, an FDA-approved agent, in a co-morbidity model of stroke that combines high age and tauopathy with thromboembolic cerebral ischemia. Following thromboembolic stroke bexarotene enhanced autophagy in the ischemic brain concomitantly with a reduction in lesion volume and amelioration of behavioral deficits in aged transgenic mice expressing the human P301L-Tau mutation. In *in vitro* studies bexarotene increased the expression of autophagy markers and reduced autophagic flux in neuronal cells expressing P301L-Tau. Bexarotene also restored mitochondrial respiration deficits in P301L-Tau neurons. These newly described actions of bexarotene add to the growing amount of compelling data showing that bexarotene is a potent neuroprotective agent, and identify a novel autophagy-modulating effect of bexarotene.

Stroke is one of the most common causes of death and disabilities worldwide^1^. Currently, the treatment of stroke relies mainly on recanalization of the cerebral blood vessels mechanically or with tissue plasminogen activator (tPa) but only if the stroke type and time course are appropriate. Although the majority of strokes occur in elderly people and the condition is typically accompanied by comorbid diseases such as dementia^2^,^3^, this is rarely taken into consideration in the design of preclinical studies aiming for novel therapies. Importantly, neurofibrillary tangles (NFT), which are aggregates of hyperphosphorylated protein tau, are a universal feature of older individual’s brains, and association of severe tau pathology with dementia lacking amyloid-β (Aβ) deposits is more prevalent than previously thought^4^,^5^. Moreover, a growing body of evidence suggests that dysfunction of the brain vascular network is a common pathological feature of neurodegenerative diseases, including diseases leading to dementia, such as Alzheimer’s disease (AD)^6^. Hyperphosphorylation of tau protein has also been reported in both animal models and human patients after cerebral ischemia, indicating a stroke-related gain of a tau-pathology related phenotype^7^,^8^. Evidence for the involvement of abnormally phosphorylated tau protein in aging and various neurodegenerative diseases, and the prion-like properties of propagating tau aggregates make it an appealing target for neurodegenerative research^9^. Counterintuitively, aging and tau pathology are rarely included in preclinical stroke research models. This may be one causative factor explaining the poor translation of preclinically successful drugs in human clinical trials.

Several studies have shown that targeting autophagy may be a therapeutic approach for stroke^10^,^11^. Autophagy is a complex process that has an important role in maintaining cellular homeostasis under physiological conditions by catabolizing cellular components such as organelles, non-functional proteins and other macromolecules. Stroke-induced damage to cellular organelles and macromolecules activates autophagy as a repair mechanism to eliminate damaged cellular components. Dysfunctional autophagy is described for several neurodegenerative diseases and stroke, yet the exact role and influence of autophagy in cerebral ischemia remains controversial. Whether the activation of autophagy is beneficial or harmful in ischemic stroke appears to depend on a delicate balance between the amount of substrate and the capacity of the autophagy machinery^11^. Nonetheless, therapeutic targeting of the autophagic cascade via pharmacological and genetic methods is protective in many preclinical models of ischemic stroke.

Bexarotene is an FDA-approved retinoid X receptor (RXR) agonist currently used for the treatment of cutaneous lymphoma^12^. Studies in different cancer models have shown that in addition to targeting the cancer related genes affecting cell proliferation, differentiation and apoptosis, bexarotene modulates protein biosynthesis and mitochondrial bioenergetics^13^. To our knowledge, the effect of bexarotene on autophagy has not previously been studied. Over the last three years bexarotene has been shown to be beneficial in models of various neurodegenerative diseases including Parkinson’s disease, amyotrophic lateral sclerosis and epilepsy^14^,^15^,^16^. Importantly, we and others have previously demonstrated that bexarotene reverses memory deficits in an AD mouse model through increasing clearance of soluble Aβ by apolipoprotein E (apoE)^17^,^18^. Bexarotene also regulates neuronal differentiation^19^. In addition, reductions in the levels of phosphorylated tau are evident in human apoE4 expressing bexarotene-treated mice^14^,^15^. Recently, bexarotene was shown to be protective in stroke models of young and healthy mice and rats with abrupt reperfusion after 30 min or 2 h of ischemia, respectively. The protection provided by bexarotene was found to be associated with modulation of the peripheral immune response^20^ in mice and reduction of blood-brain-barrier permeability in rats^21^. Although evidence supports the beneficial effects of bexarotene treatment in disorders affecting the central nervous system, the drug has not previously been tested in stroke models mimicking the most common clinical cases, i.e. thromboembolism without abrupt reperfusion^22^, taking into account the high age and tauopathy, which is commonly observed in the brains of aged individuals and in more severe form during dementia.

In this study we investigated the neuroprotective effect of bexarotene in a co-morbidity model of stroke that combines high age and tauopathy with thromboembolic cerebral ischemia. We demonstrate for the first time that bexarotene is protective in a clinically relevant murine comorbidity model and identify a novel autophagy-modulating effect of bexarotene. Our results suggest that bexarotene can be effectively repurposed for the therapy of ischemic stroke during aging and comorbid tau pathology.

## Materials and Methods

### Mice

All the experiments were approved by the national Animal Experiment Board of Finland and followed the animal protection guidelines of the Council of the European Union. A total of 97 male and female mice of 25–26 months of age expressing the P301L mutant human 2N/4 repeat Tau^23^ were used in this study. 85 wildtype (WT) littermates served as controls. Due to the high age of the animals 16% of the animals died before the end of the study. In addition, 12 animals were excluded because of age-related health-issues, 7 mice were excluded due to spontaneous reperfusion (confirmed with laser Doppler) and 4 were excluded because of visible hemorrhages in MRI. The observed deaths and exclusions occurred independently of the experimental treatment and genotype.

### Thromboembolic stroke and drug preparation

Thromboembolic stroke was induced as previously described PMID 17702959 Orset *et al.*^24^. Briefly, animals were deeply anesthetized with 5% isoflurane and the anesthesia was maintained with 1.5–2.0% isoflurane in a 70/30% mixture of N_2_O/O_2_. Rectal temperature was maintained at 36.5 ± 0.5 °C during the surgical procedures using a feedback-regulated heating system (PanLab, Harvard Apparatus, Barcelona, Spain). A small incision was made between the ear and the eye, the temporalis muscle was retracted and the skull exposed. An approximately 1 mm wide hole was drilled in the temporal bone above the middle cerebral artery (MCA). A small craniotomy was performed, the dura was excised, and the MCA was exposed. 1 μl (1U) of murine thrombin (Diagnostica Stago S.A.S, Asnières sur Seine Cedex, France) was then injected into the MCA with a hematologic micropipette (calibrated at 15 mm/μL; Assistent ref. 555/5; Hoecht, Sondheim-Rhoen, Germany). After the injection, the occlusion was confirmed and monitored with laser Doppler monitor (Moor Instruments, Devon, UK) for 30 min. After this the muscle was returned to the original place and the skin was sutured. Sham mice underwent the same surgical procedures except thrombin injection. Animals were returned to their home cages after recovering from anesthesia. Separate cohorts were used to measure blood carbon dioxide and oxygen partial pressures as well as pH with iSTAT analyzer (Abbott, Abbott Park, IL, USA).

Bexarotene (Targretin, Eisai Co. Ltd, Tokyo, Japan) or vehicle (saline) was administered to the animals by oral gavage immediately after surgery at a dose of 100 mg/kg. Justification for the dosing has been described elsewhere^25^. Subsequent administration of bexarotene occurred 1, 2, 4, 6, 8, 10 days after surgery at the same dose. Mice were sacrificed 3 or 12 days post ischemia and tissues collected as described below.

### Assesment of outcome

Multislice T2-weighted magnetic resonance imaging (MRI) images were used to quantify infarct volumes 3 days post ischemia. Mice were anesthetized with isoflurane and placed inside a vertical 9.4T Oxford NMR 400 magnet (Oxford Instrument PLC, Abingdon, UK) equipped with a quadrature volume coil. Images (repetition time 300 ms, echo time = 40 ms, matrix size = 128*256, field of view 19.2 × 19.2 mm^2^, slice thickness = 0.8 mm, and slices 12) were analyzed with in-house programmed software (Aedes) running in MATLAB (version 7.12.0.635, Mathworks, Natick, MA, USA) and the lesion volume was calculated with Shuaib’s indirect method^26^.

CatWalk gait analysis (CatWalk XT, version 10, Noldus Information Technology, Wageningen, Netherlands) was used to assess the motor outcome and functional recovery of the mice after ischemia as described^27^. Mice were tested for at least 3 uninterrupted runs 3 days before and 7 days after ischemia.

### Tissue collection

Mice were anesthetized with tribromoethanol (Avertin, Sigma-Aldrich, St. Louis, MO, USA) and transcardially perfused with heparinized (2500 IU/L) saline. A subset of the brains were fixed with 4% paraformaldehyde for 24 h at 4 °C. Another set of brains were dissected under a stereotactic microscope, and the visually determined peri-ischemic and corresponding contralateral samples were snap frozen in liquid nitrogen.

### Immunohistochemistry

Following fixation the brains were cryoprotected in 30% sucrose for 48 hours, frozen in liquid nitrogen, and cut to 20 μM sections with a cryostat (Leica Microsystems GmH, Wetzlar, Germany). Sections were blocked with 10% normal goat serum (Merck Millipore, Billerica, MA, USA) or mouse on mouse blocking reagent (Vector Laboratories INC, Burlingame, CA, USA) and let to react with primary antibodies (Iba-1, 1:250 dilution; Wako Chemicals GmbH, Neuss, Germany; GFAP, 1:500 dilution; DAKO, Glostrup, Denmark; amyloid-β, 1:1000 dilution, Covance, Princeton, NJ, USA; active cleaved caspase-3, 1:200 dilution, Cell Signaling, Danvers, MA, USA; LC3b, 1:200 dilution, Cell Signaling, Danvers, MA, USA; AT100, 1:300 dilution Fujirebio Diagnostics INC, Malvern, PA, USA; p62, 1:500 Cell Signaling Technology, Danvers, MA, USA) overnight at room temperature (RT). Aβ, caspase-3, LC3b and p62 staining required heat-mediated antigen retrieval in aqueous sodium citrate buffer pH 6.0 before application of the primary antibodies. After washing the sections with PBS containing 0.5% Tween20 (Sigma-Aldrich, St. Louis, MO, USA) Iba-1 and GFAP-treated sections were incubated for 2 hours with Alexa Fluor 568 secondary antibody (1:200 dilution, Invitrogen, Eugene, OR, USA) and embedded with Vectashield mounting medium (Vector Laboratories INC, Burlingame, CA, USA) for fluorescence with 4′,6-diamidino-2-phenylindole (DAPI). Sections treated with other antibodies were incubated with biotinylated secondary antibody (1:200 dilution, Vector, Burlingame, CA, USA) for 2 hours and thereafter reacted with avidin-biotin-complex reagent (Vector Laboratories INC, Burlingame, CA, USA). Nickel-enhanced 3,3′-diaminobenzidine (DAB) was used for visualization of the immunoreactivities.

For quantification, the immunostained 5–7 consecutive brain sections at 400 μM intervals were imaged using AX70 microscope (Olympus Corporation, Tokyo, Japan) connected to a digital camera (Color View 12 or F-View, Soft Imaging System Gmbh, Muenster, Germany) and AnalySIS Software (Soft Imaging System Gmbh, Muenster, Germany). Iba-1, GFAP, p62 and LC3b were imaged from the peri-ischemic area (cortical area immediately adjacent to the visually determined infarct border) and corresponding area on the contralateral hemisphere (Iba-1, LC3b and GFAP). Aβ and cleaved caspase-3 were imaged on the lesion site only. AT100 was imaged in the peri-ischemic cortex and contralateral hippocampus. The images were analyzed using ImagePro Plus Software (version 6.0, Media Cybernetics, Rockville, MD, USA) for the immunoreactive area.

### Primary cortical neuron culture

Primary neuron cultures were prepared from P301L-Tau transgenic (TG) and WT embryos. Cortices from E15 embryos were dissected and dissociated with trypsin (0,0125% for 15 min at 37 °C, Sigma-Aldrich, St. Louis, MO, USA). The reaction was stopped with trypsin inhibitor solution containing DNAse (Sigma-Aldrich, St. Louis, MO, USA). Neurons were dissociated, counted and plated on poly-d-lysine (Sigma-Aldrich, St. Louis, MO, USA) coated 48-, 24- (Seahorse Bioscience, MA, USA) and 6-well plates at a density of 150 000, 80 000 and 1,8 × 10^6 ^cells/well respectively, in Neurobasal media containing 2% B27, 500 μM l-glutamine and 1% penicillin–streptomycin (all ThermoFisher Scientific, Waltham, MA, USA). 50% of the medium was changed four days after plating and cultures were used for experiments on days 6–7 *in vitro*.

### N2a cell culture

Mouse neuroblastoma 2a cell line (N2a) constitutively expressing P301L-Tau and WT controls were cultured on standard cell culture dishes in Dulbecco’s Modified Eagle Medium with GlutaMAX-1 containing D-glucose (4.5 g/L) and sodium pyruvate (0.11 g/L) supplemented with 10% heat inactivated FBS and 1% penicillin-streptomycin (all ThermoFisher Scientific, Waltham, MA, USA). For experiments, cells were plated on 96- well plates for cell viability assay (8000 cells/well), on 6-well plates for Western blot (300 000 cells/well), or 12 and 24-well plates for CellROX assay (200 000 or 100 000 cells/well respectively), and treated 2 days after plating.

### Cell treatments

Treatment of cortical neurons was carried out in medium containing 50% fresh Neurobasal medium containing 2% B27, 500 μM l-glutamine and 1% penicillin–streptomycin and 50% media collected from the cells. Neurons were treated for 24 h with 0.5 μM bexarotene prepared as above and/or 400 μM glutamate (Sigma-Aldrich, St. Louis, MO, USA). For viability, cells were pre-treated for 2 h with bexarotene prior to the addition of glutamate. N2a cells were treated with growth medium without supplements containing 5 μM bexarotene.

### MTT viability assay

The MTT reduction assay was performed 22 hours after treatment with bexarotene and glutamate according to Denizot & Lang^28^ with the following modifications. Briefly, following removal of the media (3-(4, 5-dimethylthiazolyl-2)-2, 5-diphenyltetrazolium bromide) was added to cells at a concentration of 120 μM in culture media. The cells were incubated for approximately 2 hours at 37 °C at 5% CO_2_ until blue crystals were visible by eye. Following removal of the media, crystals were dissolved in DMSO. 100 μl aliquots were distributed to 96-well plate wells and the absorbances were read 585 nm with Wallac Victor^2^ 1420 multilabel counter (PerkinElmer Inc, Waltham, MA, USA). Results were calculated as relative absorbances compared to the control wells.

### Western Blotting

Tissue samples were processed for tau Western blotting by lysing peri-ischemic or contralateral cortex samples in homogenizing buffer containing 0.25 M sucrose, 10 mM Hepes, pH 7.2, 1 mM MgOAc2, 2 μM paclitaxel (Sigma-Aldrich, St. Louis, MO, USA) with 1× Complete protease inhibitor (Roche Holding AG, Basel, Switzerland) and PhosSTOP phosphatase inhibitor (F. Hoffmann-La Roche AG, Basel, Switzerland). Samples were centrifuged at 900 × *g* at 4 °C for 10 min to yield the postnuclear fraction. For autophagy marker detection tissues were lysed in RIPA buffer (50 mM TRIS, 150 mM NaCl, 1% Triton-X, 2% SDS, 0.5% sodium deoxycholate). Protein concentrations were measured from cytosolic fractions using protein assay dye reagent (Bio-Rad Laboratories Inc, Hercules, CA, USA). Samples from cell cultures were lysed and collected into SDS sample buffer (0.0625 M TRIS-HCl, 2.3% SDS, 5% β-mercaptoethanol, 10% glycerol, bromophenol blue). Equal amounts of protein were run in 10 or 15% SDS-PAGE gels and proteins were transferred to PVDF-hybond membranes (380 mA, 1 h, 4 °C). The membranes were then blocked for 30 min with 5% milk in 0.2% PBST and let to react with primary antibodies (LC3b, Cell Signaling Technology Inc, Denvers, MA, USA; p62, Cell Signaling Technology, Danvers, MA, USA; total Tau, Fujirebio Diagnostics INC, Malvern, PA, USA s; β-actin, Sigma-Aldrich, St. Louis, MO, USA; phosphospesific anti-Tau-205, ThermoFischer scientific, Waltham, MA, USA; ApoE, Santa Cruz Biotechnology Inc, Dallas, TX, USA; MMP-9, R&D Systems Inc, Minneapolis, MN, USA) diluted 1:1000 in 5% BSA in PBS-Tween20 with 0.02% NaN_3_, overnight at 4 °C. After washing three times with PBST, secondary antibodies diluted 1:2000 in blocking solution (anti-mouse, anti-rabbit or anti-sheep ECL HRP; GE healthcare life sciences, Uppsala, Sweden) were incubated for 2 hours at RT. Following a further three washes in PBST the proteins were visualized with ECL-plus kit (ThermoFischer scientific, Waltham, MA, USA) and scanned with Storm 860 scanner (Molecular dynamics, Caesarea, Israel). β-actin was diluted 1:5000 in 5% BSA in PBS-Tween20 with 0,02% NaN_3_, and detected with Cy3-conjugated anti-rabbit secondary antibody (Jackson ImmunoResearch laboratories Inc., PA, USA) and scanned with Typhoon 9400 variable mode imager (GE Healthcare life sciences, Uppsala, Sweden). Densitometry was performed with Imagequant TL software (GE Healthcare life sciences, Uppsala, Sweden). All protein expression data are shown relative to the expression of β-actin. LC3b quantification was performed according to Mizushima and Yoshimori where the level of the LC3b-II band is compared to the expression of β-actin^29^.

### Assessment of autophagic flux by LC3 turnover assay

Control and P301L-Tau expressing N2A cells were treated for 24 h with 0.5 μM bexarotene. Four hours before lysing the cells for Western blot a subset of the neurons were treated with a 0.1 μM concentration of the late stage autophagy inhibitor bafilomycin A1 (BAF; Sigma-Aldrich, St. Louis, MO, USA). Cells were collected in lysis buffer as indicated above and analyzed by Western blotting with anti-LC3 and anti-β-actin antibodies. After densitometry the autophagy flux indexes of cells were calculated as the difference in LC3II/actin ratios between samples plus and minus BAF^30^. The flux index of bexarotene-treated cells was expressed in proportion to that of control cells.

### Measurement of mitochondrial function

Mitochondrial function of primary cortical neurons was measured utilizing Seahorse XF24 extracellular flux analyzer (Seahorse Bioscience, MA, USA). The cultures were subjected to mitochondrial stress test with the sequential injection of the following compounds: oligomycin (1.26 μM), carbonyl cyanide 4-(trifluoromethoxy)phenylhydrazone (FCCP, 1 μM) and a mixture of antimycin and rotenone (both 0.5 μM) diluted in XF Assay medium supplemented with D-glucose (25 mM, all Sigma-Aldrich, St. Louis, MO, USA), sodium pyruvate (0.23 mM) and L-glutamine (0.5 mM both ThermoFisher Scientific, Waltham, MA, USA). The oxygen consumption rate (OCR, pmol/min) was measured and expressed as percentage of the basal respiration. The number of respiratory units at time zero is shown as 100%.

### Statistical methods and exclusion criteria

Animals were randomized to treatment groups using GrapPad QuickCalcs software. All the data collected from the study was analyzed blinded to the treatment groups and the statistical analysis was run with GrapPad Prim (version 5, GraphPad Software Inc, La Jolla, CA, USA) using t-test and 2-way ANOVA to compare means of interest assuming 2-sided distribution. Catwalk gait analysis data was analyzed using repeated measures 2-way ANOVA followed by Bonferroni post-test. Animals that showed visible hemorrhages in MRI images were excluded from analyses. Data from cell culture studies is shown as relative ratio compared to the control group. Data are expressed as means ± S.D. unless otherwise stated and n numbers are reported in each figure legend. Statistical significance was assumed if p < 0.05.

## Results

### Bexarotene reduces lesion size in aged P301L-Tau TG mice, but not in WT mice

**Figure 1:**
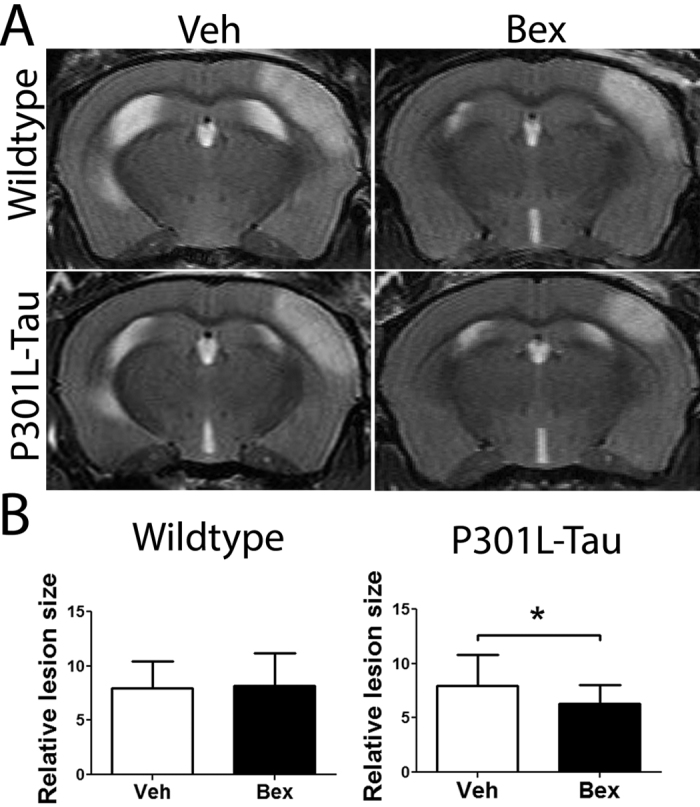
Bexarotene treatment reduces lesion size in P301L-Tau TG mice but not in WT animals. (**A**) Representative MRI images taken 72 h post ischemia. Bexarotene was administered orally at a dose of 100 mg/kg/day immediately after surgery and subsequently on days 1 and 2 after ischemia. (**B**) Lesion volumes were calculated from MRI images at 3 days post ischemia with Shuaib’s indirect method (infarct volume = [volume of left hemisphere − (volume of right hemisphere − measured infarct volume)]/volume of left hemisphere). Unpaired t-tests within genotypes, TG p = 0.048 N = 12–17/group. *p < 0.05. Veh = vehicle treated animals, Bex = bexarotene treated animals.

To determine whether bexarotene is protective in thromboembolic stroke in aged mice with tau pathology bexarotene was administered orally to mice immediately after MCA occlusion and 1, 2, 4, 6, 8 and 10 days after surgery. As visualized by MRI imaging 3 days after surgery, the thromboembolic stroke induced lesions in the motor and sensory cortical areas of the aged mouse brains (Fig. 1A). Volumetric analyses of infarct sizes revealed a significant reduction in the lesion in bexarotene-treated, aged P301L-Tau TG mice, but not in WT mice (Fig. 1B). There was no genotype-related difference between Tau-TG and WT animals in the lesion volume. The physiological parameters were unaltered by bexarotene treatment (data not shown). These data suggests that tauopathy renders the aged ischemic brain sensitive to bexaterone after thromboembolic insult.

### Bexarotene treatment protects neurons from glutamate-induced excitotoxicity *in vitro*

**Figure 2:**
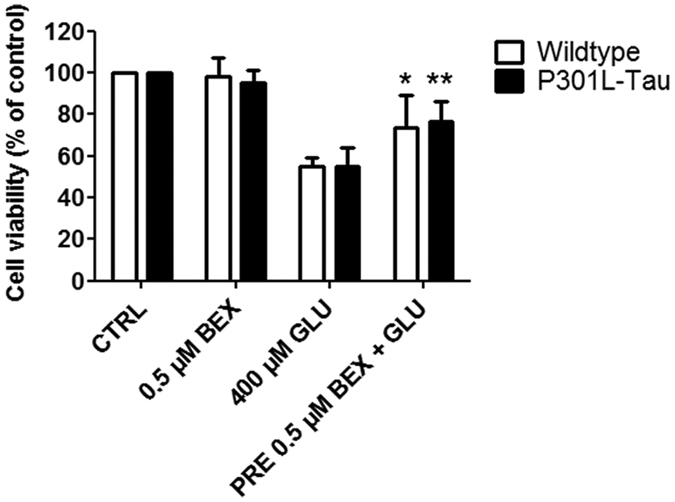
Bexarotene protects cortical neurons against excitotoxicity *in vitro*. Primary cortical neurons cultured from E15 WT and P301L-Tau embryos were treated for 2 h with 0.5 μM bexarotene prior to the addition of 400 μM glutamate. Cell viability was determined 24 h later with MTT assay. Data are normalized to the untreated, control cells. Paired t-test against CTRL, WT p = 0.044, TG p = 0.003 N = 4/group. *p < 0.05, **p < 0.01. CTRL = control, GLU = glutamate, BEX = bexarotene, PRE = pre-treatment.

To determine if bexarotene is directly neuroprotective the effect of bexarotene against excitotoxic insult was assessed by treating primary cortical neuron cultures with glutamate. These cultures contain approximately 90% neurons and 10% glial cells^31^. Treatment of neurons with glutamate for 24 h caused a 50% reduction in cell viability as measured by the MTT assay (Fig. 2). A 2 h pre-treatment with bexarotene significantly improved the viability of cortical neurons treated for 24 h with glutamate. Bexarotene was protective against glutamate toxicity to a similar extent in both control and P301L-Tau neurons. Neuronal viability was not affected by treating cells with bexarotene alone. These results demonstrate that bexarotene can have direct protective effects against glutamate-induced neurotoxicity independent of tauopathy.

### Motor recovery is impaired in aged P301L-Tau TG mice after stroke but improved by bexaterone treatment

**Figure 3:**
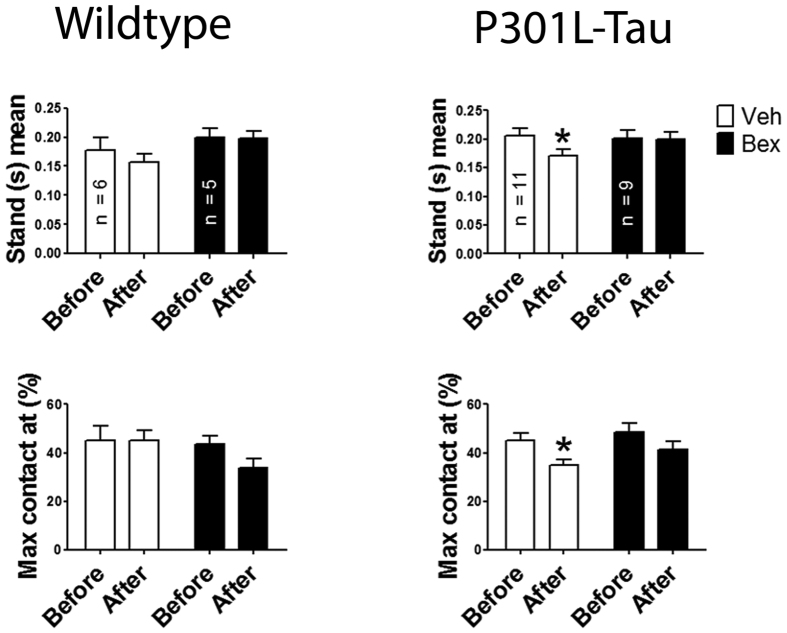
Bexarotene has a beneficial effect on recovery of motor coordination in ischemic P301L-Tau TG mice. CatWalk gait analysis, ‘stand mean’ values of right side hind limb expressed as seconds and ‘max contact at’ as a percentage 3 days before and 7 days after stroke. N = 5–11 group. Data are shown as means ± SEM. *p < 0.05 in post-tests within treatment group.

To determine, if in addition to being directly neuroprotective, bexarotene treatment can have beneficial effects on the motor recovery of ischemic mice we applied CatWalk gait analysis to evaluate motor function at 7 days after stroke. Both WT and P301L-Tau TG animals displayed few stroke-induced changes in gait analyses, most likely because of diminished motor performance, which has been reported to occur with aging^32^,^33^. In comparison to WT mice, there was a consistent reduction in the right hind limb Stand mean and Max contact at -values in the P301L-Tau TG mice with ischemic stroke. These effects were ameliorated in mice receiving bexarotene treatment (Fig. 3). Stand mean value means step duration for each limb in seconds. Max contact at value refers to the point where the braking phase of the gait turns into the propulsion phase. Both parameters remained unaffected in WT mice and in sham mice after the surgery regardless of the treatment group. The slight alteration the P301L-Tau TG mice show in the right hind limb use as well as in the transition from the braking to the propulsion phase may have been caused by the ischemic damage in the sensory and motor cortex areas, which in turn leads to the improper timing of limb use during the step cycle. These results suggest that bexarotene has a slight, but significant beneficial effect on the motor performance of the aged, P301L-Tau TG mice 7 days after the insult.

### The levels of tau, MMP-9, Aβ burden, gliosis and the peripheral immune response are not altered in bexarotene-treated ischemic P301L-Tau TG mice

**Figure 4:**
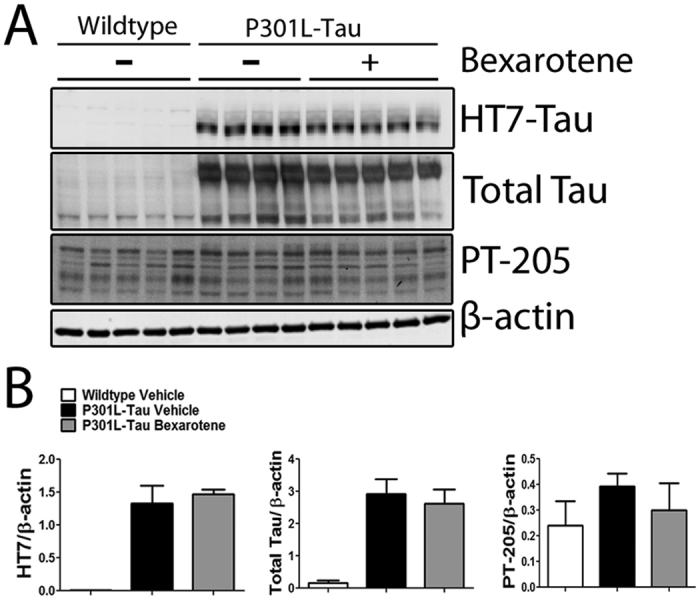
Bexarotene treatment does not reduce total or phosphorylated tau in ischemic P301L-Tau TG brains. (**A**) Representative images of Western blots where total and phosphorylated tau protein were visualized from the peri-ischemic brain area of bexarotene -and vehicle treated ischemic mice. Total protein fractions were separated with gel electrophoresis and probed with antibodies against total human tau protein (HT7 and total Tau) and tau phosphorylated at Thr205 (PT-205). Samples from wildtype animals are shown as controls. (**B**) The bands were quantified and normalized to β-actin. Analysis with unpaired t-tests between vehicle and bexarotene treated TG animals. N = 4–5/group.

The P301L mutation is associated with aggregation of tau and neuronal loss in mice^23^. To assess whether bexarotene mediates its protective effects through modulation of tau protein expression or phosphorylation, we performed Western blot analysis for peri-ischemic cortical samples collected from the aged ischemic animals. The extent of tau pathology was detected with antibodies against total tau and tau phosphorylated at Thr205. Bexarotene treatment did not significantly affect either the levels of total human tau or the Thr205 phosphorylated form of tau 12 days after stroke (Fig. 4). A similar lack of effect was seen at 3 days post ischemia (data not shown). In addition, tau phosphorylated at Ser212 and Thr214 was not affected by bexarotene treatment as measured by immunoreactivity following AT100 staining in the peri-ischemic area (Fig. S1).

Bexarotene is known to reduce the levels of Aβ by increasing clearance through ApoE and to influence the inflammatory status of the brain^34^. Quantification of immunohistological stainings revealed that bexarotene failed to reduce pan-Aβ immunoreactivity in the brain (Fig. S2) even though the increase in ApoE was detected by Western blotting (data not shown). A similar lack of effect was seen in the extent of activation of astrocytes and microglia in the peri-ischemic area of the brain as detected by immunostaining against GFAP and Iba-1, respectively (Fig. S2). Furthermore, bexarotene treatment did not have an effect on MMP-9 levels in the peri-ischemic area (Fig. S2).

A recent report by Certo *et al.* demonstrated that bexarotene modulates the peripheral immune response and increases the number of neutrophils infiltrating the ipsilateral hemisphere of young healthy mice after transient ischemia^20^. To determine whether bexarotene has the same effect in aged mice after thromboembolic stroke, flow cytometry was applied to determine spleen alterations, and neutrophil infiltration into the brain was assessed by immunohistochemistry. FACS analyses revealed that the number of Ym-1 positive neutrophils was not altered in the spleens of bexarotene-treated mice at 3 days post-ischemia (Fig. S3). Similarly, the number of Ym1 positive neutrophils in the brain sections was not altered by bexarotene treatment of the P301L-Tau TG mice 1 day after stroke indicating that peripheral neutrophils do not have a major role in neuroprotection in aged mice (Fig. S4). Spleen weights were also not altered by bexarotene-treatment (data not shown).

### Bexarotene prevents the ischemia-induced reduction in LC3B levels in the peri-ischemic brain area of P301L-Tau TG mice

**Figure 5:**
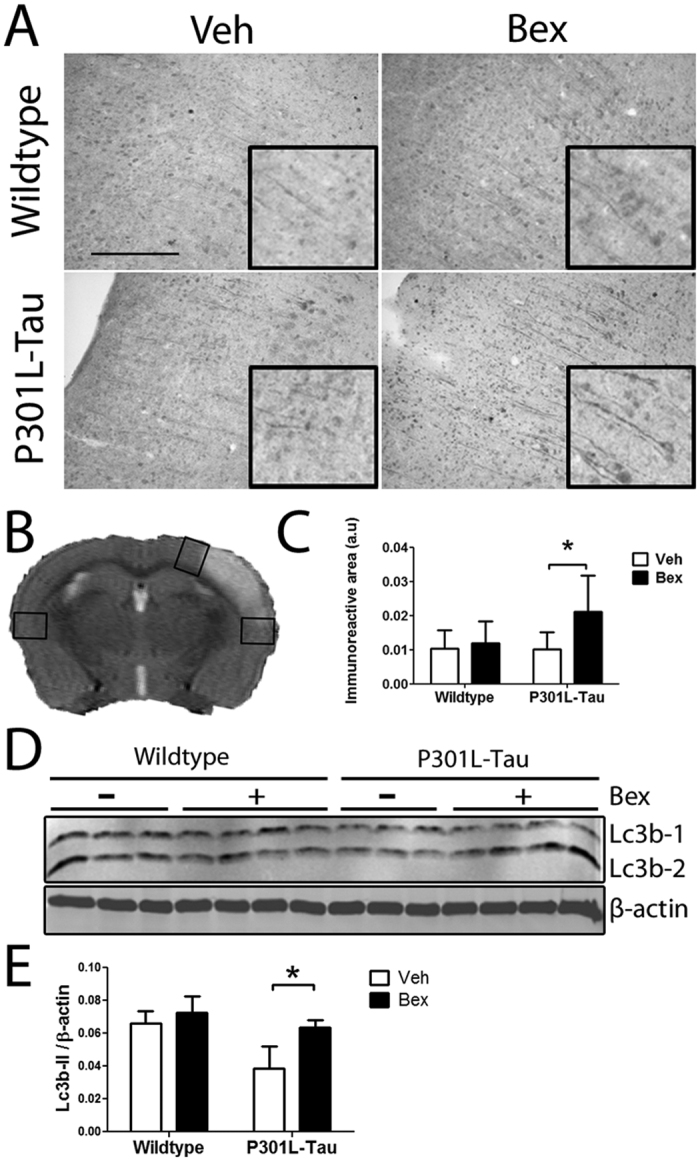
LC3B protein levels are increased by bexarotene treatment in the peri-ischemic area of P301L-Tau TG mouse brains after stroke. (**A**) Representative images showing LC3B immunoreactivity in the peri-ischemic area of WT and P301L-Tau TG mice at 12 days post-stroke. The boxes show select areas of the immunoreactivity at high magnification (**B**) LC3B immunoreactivity was quantified from three distinct brain areas represented by the rectangles. Analysis with 2-way ANOVA with Bonferroni post-tests within genotypes (**C**) The LC3B positive immunoreactive area was quantified from the peri-ischemic brain region. N = 7–8/group. (**D**) Representative image of LC3B Western blots run from samples of the peri-ischemic brain area of mice treated with bexarotene or vehicle 3 days after stroke. (**E**) Densitometry of LC3B-2 expression relative to β-actin. Analysis with unpaired t-test against vehicle, TG p = 0.031 N = 3–4/group. *p < 0.05. Scale bar 100 μM. a.u. = arbitrary unit.

Bexarotene is in clinical use for the treatment of cutaneous lymphoma where its mechanism of chemopreventive actions is likely connected to the transcriptional modulation of genes responsible for cell proliferation, differentiation and apoptosis^13^. Thereby we next focused our efforts on deciphering whether bexarotene-mediated protection in aged P301L-Tau TG mice is associated with alterations in apoptosis or autophagy. Bexarotene treatment did not alter the immunoreactivity of cleaved caspase-3 in the peri-ischemic area, indicating that the drug is not likely exerting its beneficial effect by preventing apoptosis (data not shown). We then applied LC3B immunostaining and Western blot to determine the extent of autophagy in the post-ischemic brain sections (Fig. 5). The LC3B staining pattern was diffuse and homogenously distributed in the vehicle treated mice (Fig. 5A). The degree of LC3B staining was not affected by the presence of the P301L Tau mutation in vehicle-treated animals (Fig. 5C). Importantly, LC3B-immunoreactivity in the peri-ischemic area of the P301L-Tau TG mice was higher in bexarotene treated mice compared to vehicle treated mice. A corresponding difference was not observed in WT mice. This difference in TG mice was observed specifically in LC3B staining in cellular processes. Bexarotene did not affect LC3B staining in the contralateral hemisphere (data not shown). To confirm the observed difference in LC3B immunostaining we next applied Western blotting to assess the levels of LC3B in brain homogenates of the peri-ischemic area (Fig. 5D). We observed that the peri-ischemic levels of LC3B-II were decreased in P301L-Tau TG mice when compared to the levels in WT mice. However, bexarotene treatment prevented this reduction of LC3B-II in P301L-Tau TG mice but had no effect on corresponding LC3B-II levels in WT mice (Fig. 5E). Bexarotene did not affect the expression of the autophagy-related markers p62 or Beclin-1 (data not shown). Taken together, these data indicate that bexarotene treatment prevents the reduction of LC3B-II protein levels observed in aged, ischemic P301L-Tau mouse brains concomitantly with a reduction of lesion volume and amelioration of behavioral deficits.

### Bexarotene increases the expression of autophagy markers and reduces autophagic flux in neuronal cells expressing P301L-Tau

**Figure 6:**
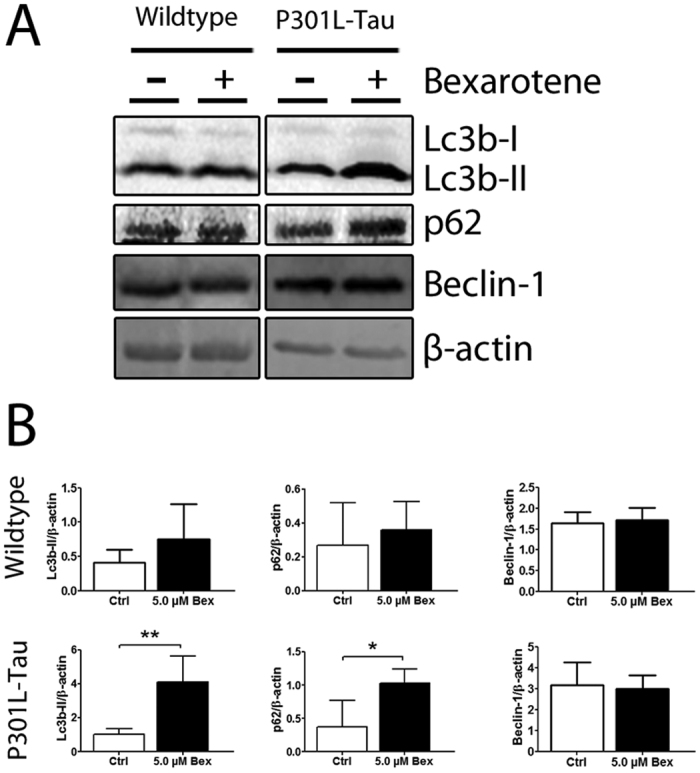
Bexarotene treatment increases the levels of autophagy markers LC3B-II and p62 in P301L-Tau neuronal cells. (**A**) Representative Western blots showing expression of LC3B, p62 and beclin-1 after 24 h treatment of N2A cells with 0.5 μM bexarotene. (**B**) Densitometry results of autophagy markers after normalization to β-actin. Paired t-test against Ctrl, LC3B TG p = 0.005, p62 TG p = 0.023 N = 6/group. *p < 0.05, **p < 0.01.

Given the observed alterations of LC3B in bexarotene-treated P301L-Tau TG mice we next sought to characterize the potential effects of the drug on the autophagic pathway in a homogenous cell population *in vitro*. To do this, neuronal N2a cells carrying the same P301L-Tau mutation were treated with non-toxic concentrations of bexarotene for 24 h, after which the expression of autophagy markers were assessed by Western blotting (Fig. 6). Tau levels were not altered by bexarotene treatment (data not shown). As seen *in vivo*, the levels of LC3B-II protein were higher in bexarotene treated than vehicle treated P301L-Tau neuronal cells, whereas no corresponding difference was seen in normal N2a cells. Similarly, p62 protein levels were higher after bexarotene treatment in P301L-Tau cells. P62 is one of the specific substrates that are selectively degraded through the autophagy pathway and serves as a link between LC3 and ubiquitinated substrates. The concurrent increase of this marker with LC3B-II suggests that bexarotene causes an accumulation of autophagic vesicles at least in part by impairing autophagosome degradation.

**Figure 7:**
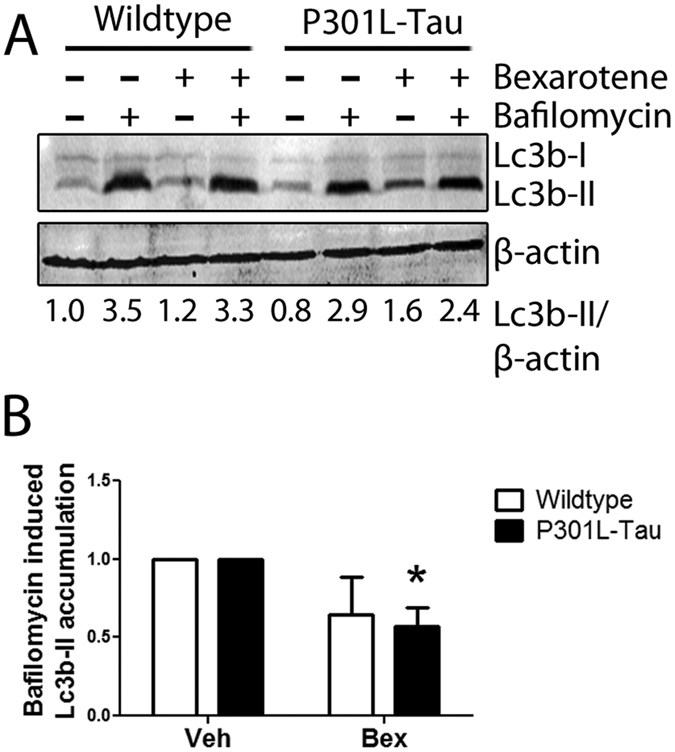
Bexarotene treatment modulates autophagic flux in neuronal cells carrying the P301L-Tau mutation. (**A**) Representative LC3B and β-actin Western blot images of N2A cells treated for 24 h with 0.5 μM bexarotene in the presence or absence of BAF. BAF was added to the cells at a concentration of 0.5 μM for 3 h prior to lysing the samples. (**B**) Autophagic flux indexes calculated from densitometry data illustrated as the difference in LC3B-II ratios between samples plus and minus BAF and expressed in arbitrary units (a.u.). Paired t-test against control, TG p = 0.025 N = 3/group.

To further understand whether the observed increase in LC3B-II and p62 expression was related to enhanced formation or reduced clearance of autophagosomes we next assessed the rate of autophagosome formation using the autophagic flux assay^35^. Autophagic flux is the rate at which material is cleared from the cell by autophagy and can be quantified by measuring LC3B-II turnover. Autophagic flux in bexarotene-treated N2A cells was determined by LC3B-II turnover using Western blot in the presence and absence of the lysosomal degradation inhibitor bafilomycin A1 (BAF) (Fig. 7). The autophagic flux indexes were calculated as the ratio in the amount of LC3B-II in the presence and absence of BAF, thereby representing the amount of LC3B-II-positive autophagosomes degraded through the lysosome during the BAF treatment. BAF induced a robust increase in the levels of LC3B-II in both control and P301L-Tau cells. Treatment with bexarotene caused a slight reduction in the autophagic flux index in control cells (Fig. 7B). This reduction was approximately 3-fold larger in the P301L-Tau expressing cells. These results indicate that the rate at which material is degraded by autophagy in bexarotene-treated control and P301L-Tau neuronal cells is different. It suggests that when compared to control cells, autophagy flux is reduced in bexarotene-treated P301L-Tau cells.

### Bioenergetic functions of P301L-Tau neurons are restored with bexarotene treatment

**Figure 8:**
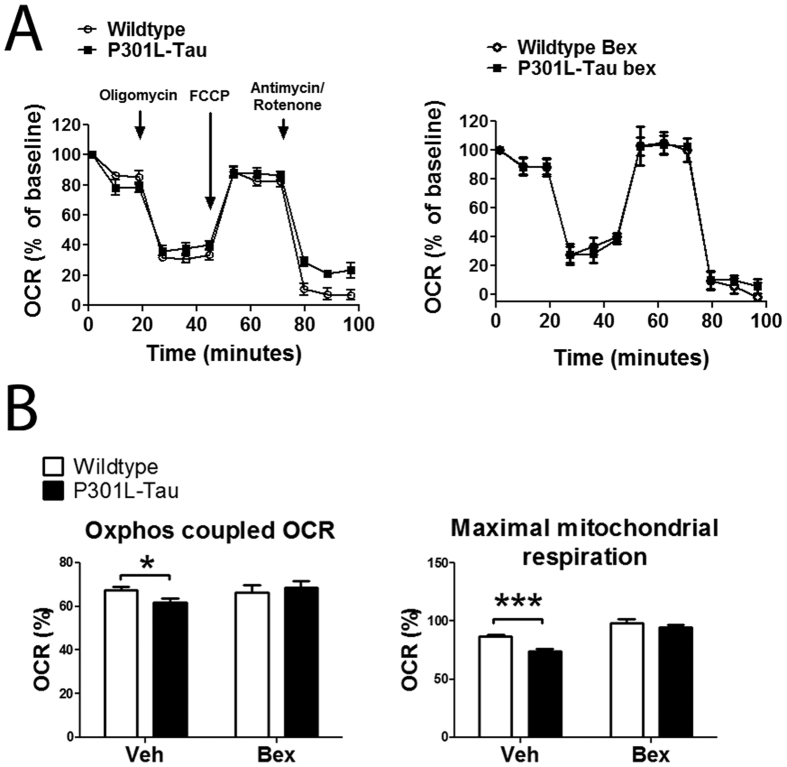
Bexarotene treatment restores mitochondrial function of P301L-Tau neurons *in vitro*. (**A**) Left panel: Traces of oxygen consumption rates (OCR) of WT and P301L-tau cortical neurons as measured with Seahorse XF analyzer by sequential additions of mitochondrial effectors at time points indicated by arrows. Right panel: OCR traces of WT and P301L-tau cortical neurons treated for 24 h with 0.5 μM bexarotene prior to analysis. (**B**) Percentages of Oxphos-coupled OCR and mitochondrial maximal respiration OCR normalized to baseline at time zero. Unpaired t-test between genotypes, oxphos-coupled OCR p = 0.019, mitochondrial maximal respiration p < 0.001 N = 4/group. Data are shown as means ± SEM. *p < 0.05, ***p < 0.001.

Autophagy is a central pathway in the removal of damaged cell organelles, including mitochondria. Furthermore, autophagy is known to affect cellular respiration^36^. To determine whether the bexarotene-induced alterations in autophagy were reflected in mitochondrial function we measured the oxygen consumption rate (OCR) of bexarotene-treated WT and P301L-Tau neurons using Seahorse XF technology. Primary cortical neuron cultures underwent measurement of the OCR using the mitochondrial stress test. After oligomycin (ATP-synthase/complex V inhibitor) injection the respiration rate of P301L-Tau neurons remained at a higher level compared to WT neurons (Fig. 8A), thereby indicating that the transgenic neurons exhibit a reduced oxidative phosphorylation (Oxphos)-coupled OCR. Following administration of rotenone and antimycin (complex I and III inhibitors) the respiration rate of P301L-Tau neurons again remained at a higher level compared to WT neurons (Fig. 8A). When neurons were treated for 24 h with 0.5 μM bexarotene prior to the mitochondrial stress test, the observed genotype-induced reductions in the OCR of P301L-Tau neurons were mitigated (Fig. 8B). These data indicate that bexarotene restores the mitochondrial respiration deficit observed in P301L-Tau neurons.

Alterations in energy metabolism are known to be associated with changes to the amount of free radicals that are generated. To assess whether bexarotene-mediated correction of the observed respiration deficit in P301L-Tau neurons was associated with a reduction in reactive oxygen species (ROS), cells were stained with the CellROX green reagent for oxidative stress detection in mitochondria and nuclei, and analyzed by flow cytometry. There was no genotype difference in the amount of basal ROS in the WT or mutant cells. As expected, treatment of both WT and P301L-Tau mutant cells with the oxidant TBHP induced a significant increase in the amount of ROS (Fig. S5). While the anti-oxidant NAC reduced ROS production induced by TBHP treatment (data not shown), bexarotene did not (Fig. S5). This data indicates that bexarotene does not reduce oxidative stress in the mitochondria as measured by the CellROX assay.

## Discussion

Discovery of novel, effective therapies for stroke is hampered by the fact that high age, primary age-related tauopathy or comorbidities are usually not taken into account in preclinical studies, which predominantly use young, healthy rodents. Moreover, while the suture model of transient MCA occlusion serves as a useful model to investigate ischemia-reperfusion pathology of stroke, additional animal models need to be used in order to obtain more reliable and perhaps more mechanistically relevant or translational information about ischemic stroke and potential therapeutic approaches. This study was undertaken to contribute to the search of new therapies for ischemic stroke. Our results indicate for the first time that bexarotene is protective in a clinically relevant murine model involving aging, thromboembolic stroke and tauopathy. We also demonstrate a novel autophagy-modulating effect of bexarotene in neuronal cells and report that bexarotene restores defective mitochondrial respiration in neurons carrying the P301L-tau mutation.

Bexarotene is FDA-approved for the treatment of cutaneous lymphoma, and has been shown to be protective in a wide variety of brain diseases^14^,^15^,^16^. Recently published studies have demonstrated that bexarotene is protective in young, healthy male rats subjected to cerebral ischemia-reperfusion injury^21^ and in young, healthy male mice subjected to transient occlusion of the MCA^20^. Our findings are consistent with previous evidence demonstrating that bexarotene reduces ischemia-induced brain damage. However, our results demonstrate that upon thrombin-induced occlusion of the MCA bexarotene is effective in aged mice only in the presence of tauopathy. A previous report has shown that the degree of ischemic damage is reduced in young P301L-Tau transgenic animals subjected to hypoxia/ischemia injury^37^, yet in our study utilizing aged mice the overexpression of human mutant tau did not affect ischemia-induced brain damage. These altered responses are most likely related to the different models used, especially with relation to age and ischemia model. Our result suggests that even though the magnitude of ischemic damage was unaltered between WT and P301L-Tau mice, the tau mutant mice are more amenable to the therapeutic effects of bexarotene. The fact that the genotype-independent protective effect was observed in primary cortical cultures exposed to glutamate suggests that while bexarotene can be directly neuroprotective during excitotoxicity *in vitro*, in the complex *in vivo* setting of aging, ischemia and tauopathy the drug modulates a distinct mechanism or pathway that is altered in these conditions. Our findings together with the previously published results highlight two important facts related to unsuccessful clinical trials: 1) the drug efficacy may be seen only in a subpopulation of stroke patients and 2) aging and the fundamental differences in preclinical models may have a pronounced impact on the outcome of drug effects.

In line with the observed reduction of ischemia-induced cell loss in aged P301L-Tau mice we also report a slight, but significant beneficial effect of bexarotene on recovery of motor coordination. Again, this effect was only observed in the P301L-Tau transgenic mice, thereby suggesting that during ischemia and aging bexarotene targets a factor specific for conditions involving tauopathy. Interestingly, the behavioral outcome was not coupled to the lesion volume data in vehicle-treated mice: the degree of ischemic damage as measured by MRI imaging was not affected by tauopathy, yet tauopathy resulted in impaired recovery of motor performance, thereby indicating that the P301L-Tau mice are relatively more inclined to display functional deficits following brain ischemia. Although the correlation between ischemic lesion volume and motor phenotype has not been assessed in detail in the thromboembolic model used in this study, it is known that infarct size correlates only modestly with outcome in stroke patients^38^. In fact, a good neurological outcome is a major recovery indicator for stroke patients regardless of the signs of brain damage in imaging studies^39^. Bexarotene treatment reduced both lesion volume and increased functional recovery during tauopathy, although it should be noted that because motor coordination is highly affected by aging^32^,^33^,^40^, the results of motor coordination testing in this study may be confounded by the older age of the animals and should be interpreted with caution. Regarding aged mice, a recent study by Tachibana *et al.* showed that long term bexarotene treatment has beneficial effects during aging by modulating synaptic proteins *in vivo*, but also causes activation of glial cells and loss of body weight^41^. During the 12-day duration of our study we did not observe any adverse side effects with bexarotene treatment, including weight loss. This suggests that in contrast to chronic diseases, clinical, short-term application of bexarotene in acute conditions such as ischemic stroke may be a feasible approach, while prolonged administration may require careful consideration of drug dosage.

The obvious culprit to consider in understanding the genotype-related difference in bexarotene response in this model is the tau protein. Several compounds have been shown to modulate tau phosphorylation during ischemia and regulation of tau is considered a therapeutic target for ischemic stroke^42^,^43^,^44^. Our data show that the beneficial effects of bexarotene were not mediated through alterations in the levels of total or phosphorylated tau, or through the well-described effect of bexarotene on reduction of Aβ^17^ that may accumulate upon aging. It has been reported that tau pathology affects remodeling of cerebral arteries^45^ and that bexarotene modulates the permeability of the blood brain barrier (BBB) through regulation of MMP-9 activity^21^. Although we cannot exclude the possibility that bexarotene-induced protection is due to BBB permeability changes in ischemic P301L-Tau mice, the fact that we did not observe any changes in MMP-9 levels in the ischemic brains suggests that this may not be the main mechanism of bexarotene action in the aged P301L-Tau mice. Moreover, bexarotene-mediated protection in aged P301L-Tau TG mice was not associated with alterations in apoptosis despite the reported impact of bexarotene on transcriptional regulation of genes responsible for cell proliferation, differentiation and apoptosis^13^.

Instead, bexarotene ameliorated the ischemia-induced deficit of P301L-Tau mice in the protein expression of LC3B, and more precisely LC3B-II, a marker for autophagy. Autophagy is a catabolic process that involves the sequestration, packaging and delivery of cellular material to the lysosomes for degradation^46^. Dysfunctional autophagy is implicated in neuronal loss in both acute and chronic neurodegenerative diseases^47^, including ischemic stroke. For example, transient hypoperfusion in the 3xTg-AD mouse model harboring the P301L-Tau mutation has been demonstrated to activate autophagy^48^. Even though targeting autophagy has been proposed as a therapeutic target for stroke^49^, studies on agents that modulate autophagy that could be used in a clinical context of stroke are still limited.

LC3B-II levels directly correlate with the number of autophagosomes and are considered one of the most reliable markers for the study of autophagy^35^. We report a significant reduction in LC3B protein levels in aged, ischemic P301L-Tau brains, which occurs concomitantly with a reduction in infarct volume. Previous studies corroborate a relationship between the degree of ischemic damage and LC3B-II protein levels^50^. Because the P301L-Tau mutation causes tau to become aggregation-prone^23^, it is possible that the mutated tau influences autophagy-mediated degradation due to the existence of increased numbers of protein aggregates. Importantly, we discovered that bexarotene treatment prevents the reduction of LC3B-II protein in ischemic P301L-Tau TG brains and brings it to similar levels as observed in WT animals, concomitantly with a neuroprotective effect. Similarly, bexarotene increased the protein levels of LC3B-II in P301L-Tau, but not control neuronal cells *in vitro*. Although modulators of autophagy have been demonstrated to influence tau expression in neuronal cells overexpressing human P301L-Tau^51^, we did not detect bexarotene-mediated alterations in tau protein expression *in vitro* or *in vivo*. Taken together, these results raised the question of whether the bexarotene-mediated increase in LC3B-II protein levels during tauopathy was the result of altered autophagosome synthesis or autophagosome turnover.

Concurrently with increased LC3B-II protein expression bexarotene treatment increased p62 levels in P301L-Tau neuronal cells. We thereby suspect that during tauopathy bexarotene causes an accumulation of autophagic vesicles at least in part by reducing autophagosome degradation. Furthermore, results from the autophagy flux assay demonstrated that bexarotene causes a 3-fold larger reduction in autophagic flux in P301L-Tau cells when compared to WT cells. Taken together, these results suggest that the observed increase of LC3B-II in bexarotene-treated P301L-Tau cells is due to a reduction in the rate at which cellular material is degraded by autophagy. Although we did not detect altered tau expression in bexarotene-treated samples we can hypothesize that the presence of tau causes an increase in the rate of autophagy (autophagosome turnover) in an attempt to clear this pathological protein. In the presence of bexarotene, tau aggregation is reduced and the rate of autophagy is slowed down. Because autophagy can be activated either in a cytoprotective or cytodestructive mode depending largely on the type and length of insult^52^, the precise influence of this process remains unclear.

Activation of autophagic pathways is associated with alterations in mitochondrial function^53^ and bexarotene has been shown to affect oxidative phosphorylation genes in dopamine neurons^54^. To determine if bexarotene treatment alters mitochondrial function we measured oxygen consumption rates in WT and P301L-Tau neurons. As has been previously reported^55^,^56^,^57^, our results demonstrated that the P301L-Tau mutation is associated with mitochondrial changes. Both ATP production and maximal respiration were reduced in P301L-Tau neurons independently of influencing mitochondrial ROS production. These defects were ameliorated with bexarotene treatment, thereby suggesting that bexarotene modulates bioenergetic functions of P301L-Tau neurons. While the exact mechanism via which bexarotene restores mitochondrial respiration deficits remains unknown, induction of the AMPK pathway, a master regulator of energy production, known to be induced by bexarotene during myocardial hypertrophy^58^, may play a role.

In summary, the results described here demonstrate that bexarotene alters autophagy in the ischemic brain concomitantly with a reduction in lesion volume and amelioration of behavioral deficits in aged mice with tauopathy after thromboembolic stroke. This novel action of bexarotene adds to the growing amount of compelling data showing that bexarotene is a potent neuroprotective agent. Because dysregulation of autophagy is observed in several neurodegenerative conditions, our results call for further studies to investigate whether bexarotene modulates the autophagic process in these diseases.

## Additional Information

**How to cite this article**: Huuskonen, M. T. *et al.* Bexarotene targets autophagy and is protective against thromboembolic stroke in aged mice with tauopathy. *Sci. Rep.* **6**, 33176; doi: 10.1038/srep33176 (2016).

## Supplementary Material

Supplementary Information
