# Supplementary material for: Bexarotene targets autophagy and is protective against thromboembolic stroke in aged mice with tauopathy
Source: Sci Rep. 2016 Sep 14;6:33176. doi: 10.1038/srep33176 (PMC5021977; doi:10.1038/srep33176)

**Bexarotene targets autophagy and is protective against thromboembolic stroke in aged mice with tauopathy**

Mikko T Huuskonen^1^, Sanna Loppi^1^, Hiramani Dhungana^1^, Velta Keksa-Goldsteine^1^, Sighild Lemarchant^1^, Paula Korhonen^1^, Sara Wojciechowski^1^, Eveliina Pollari^1^, Piia Valonen^1^, Juho Koponen^1^, Akihiko Takashima^2^, Gary Landreth^3^, Gundars Goldsteins^1^, Tarja Malm^1^, Jari Koistinaho^1^, Katja M Kanninen^1^*.

**SUPPLEMENTARY MATERIALS AND METHODS**

*Flow cytometry for neutrophils*

Spleens were harvested on ice in HBSS (Lonza). Single cell isolations were prepared by crushing tissue through 70 μm cell strainers (BD Biosciences, San Jose, CA USA), centrifuging 450 x *g* for 5 min and resuspending pellet in 2 ml of 1X BD PharmLyse (BD Biosciences, San Jose, CA USA) to lyse red blood cells. Samples were incubated for 8-10 min at RT, washed and slowly frozen at -70°C in a mixture of 90% FBS (ThermoFisher Scientific, Waltham, Massachusetts, USA) and 10% DMSO (Sigma-Aldrich, St. Louis, MO, USA). One million cells from each mouse was stained for viability using Zombie NIR (1:1000, BioLegend, San diego, CA, USA) for 15 min at RT, blocked with FcR CD16/32 antibody (1:100, clone 24G.2, BD Biosciences, San Jose, CA USA), and surface stained with Ly6G PerCP-Cy5.5 (1:75, clone 1A8, BD Biosciences, San Jose, CA USA) and Ly6B.2 FITC (1:200, clone 7/4, Bio-Rad Laboratories Inc, Hercules, CA, USA) for 30 min at 4°C. Cells were washed twice in PBS and resuspended in 1X fix/perm buffer (FoxP3 staining buffer kit, eBioscience Inc, San Diego, CA, USA) for 45 minutes at 4°C. Following washing in 1X perm wash (FoxP3 staining buffer kit eBioscience Inc, San Diego, CA, USA) cells were intracellularly stained with YM-1 (1:50, Stemcell Technologies, Vancouver, British Columbia, Canada) for 30 min at 4°C. Cells were then washed twice in 1X perm wash and stained with secondary anti-rabbit PE (1:500, ThermoFisher Scientific, Waltham, Massachusetts, USA) for 30 min at 4°C. Following two washes in 1X perm solution samples were resuspended in HBSS + 1% FBS. Data were acquired on a FACSAriaIII equipped with 488nm and 633nm lasers with standard filter setup. Analysis was performed using FCS Express ver5 (DeNovo Software, Los Angeles, CA).

*Immunohistochemistry of peri-ischemic neutrophils*

For the quantification of peri-ischemic Ym1 and Ly6G positive neutrophils 8 12-16 month old P301L- TG mice underwent ischemic surgery as previously described 1 . Half of the animals received vehicle and the rest bexarotene treatment as described above. Mice were sacrificed 24 h after ischemia and the brains were collected for immunohistochemical analysis as described above. Brain sections were probed with antibodies against Ym1 (1:100, STEMCELL Technologies, Vancouver, British Columbia, Canada) and Ly6G (1:100, BioLegend, San diego, CA, USA) and detected with Alexa Fluor 488 and 568 antibodies. Sections were imaged with Zeiss Axio Observer.Z1 microscope (Carl Zeiss AG, Oberkochen, Germany).

*Analysis of ROS production with CellROX assay*

WT and P301L-Tau expressing N2A cells were grown as stated above and treated for 24 h with 0.5 or 5 µM bexarotene in serum–free media. Assay controls were incubated for 1 hour at 37°C, 5% CO_2_, with the antioxidant, N-acetylcysteine (NAC) (10mM, Molecular Probes) then with or without a ROS inducer, tert-butyl hydroperoxide (tBHP) (200μM, Molecular Probes) for 1 hour. CellROX Green (300nM, Molecular Probes) was added to the wells for 30 min. The cells were dissociated by pipetting, washed with PBS, resuspended in PBS and incubated with viability dye SYTOX red (1:2000, Molecular Probes). Data were acquired on FACSAriaIII (BD Biosciences, San Jose, CA USA) and analyzed using FCS Express ver 5 (DeNovo Software, Los Angeles, CA, USA).

**SUPPLEMENTARY FIGURE LEGENDS**

F*igure S1: Bexarotene treatment does not affect tau phosphorylation at Ser212 and Thr214.* The AT100-positive immunoreactive area was quantified from the cortical peri-ischemic brain region and contralateral brain area of vehicle and bexarotene treated WT and P301L-tau TG mice at 12 days post ischemia. N= 7-8 /group.

*Figure S2: Bexarotene treatment does not affect Aβ, gliosis or MMP-9.* (A) The pan-Aβ positive immunoreactive area was quantified from the peri-ischemic brain region of vehicle and bexarotene treated WT and P301L-tau TG mice at 12 days post ischemia. (B) The GFAP-positive immunoreactive area was quantified from the peri-ischemic brain region of vehicle and bexarotene treated WT and P301L-tau TG mice at 12 days post ischemia. (C) The Iba-1 positive immunoreactive area was quantified from the peri-ischemic brain region of vehicle and bexarotene treated WT and P301L-tau TG mice at 12 days post ischemia. Data are shown as immunoreactive area. a.u.=arbitrary unit. N= 7-8 /group (D) MMP-9 was detected with Western blotting from the peri-ischemic area 72 h after stroke. N= 3-4/group.

*Figure S3:* *Bexarotene treatment does not affect the number of Ym-1 positive spleen neutrophils.*

Flow cytometric data from spleen samples 72 hours after stroke. (A) Representative plots showing live gated neutrophils double positive for markers Ym1 and Ly6B.2.  (B) Quantified results from Ym1, Ly6B.2. and Ly6G live gated events showing no significant bexarotene mediated effect. N=6-8/group, p>0.05 for all markers within genotypes.

*Figure S4:* *Bexarotene treatment does not affect the number of peri-ischemic Ym-1 positive neutrophils.* (A) Ly6G immunoreactive area was quantified from the peri-ischemic brain region of vehicle and bexarotene treated P301L-tau TG mice at 24 h post ischemia. (B) Ym1 immunoreactive area was quantified from the peri-ischemic brain region of vehicle and bexarotene treated P301L-tau TG mice at 24 h post ischemia. Data are shown as immunoreactive area. a.u.=arbitrary unit. (C) Number of double positive cells for Ly6G and Ym1 was counted from the peri-ischemic brain region of vehicle and bexarotene treated P301L-tau TG mice at 24 h post ischemia. N=4/group.

*Figure S5: Bexarotene treatment does not affect the amount of ROS in N2A cells.*

ROS quantification from WT and P301L-Tau expressing N2A cells by CellROX flow cytometry. Cells were treated with 0.5 μM bexarotene for 24 h prior to measurement. 200 μM TBHP was added 1 h before analysis. The data were normalized to control cells. N=4/group. *p<0.05, **p<0.01.

**SUPPLEMENTARY FIGURES**

Figure S1


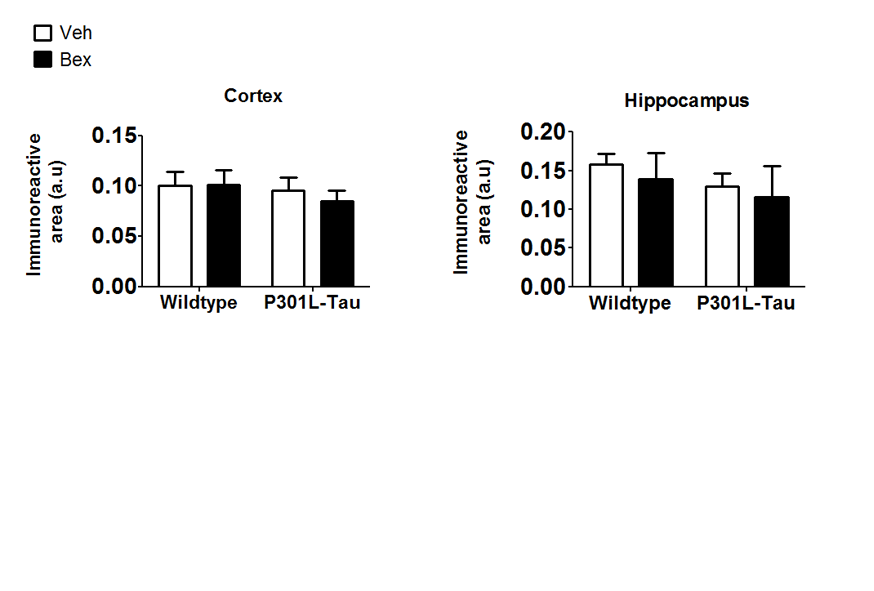


Figure S2


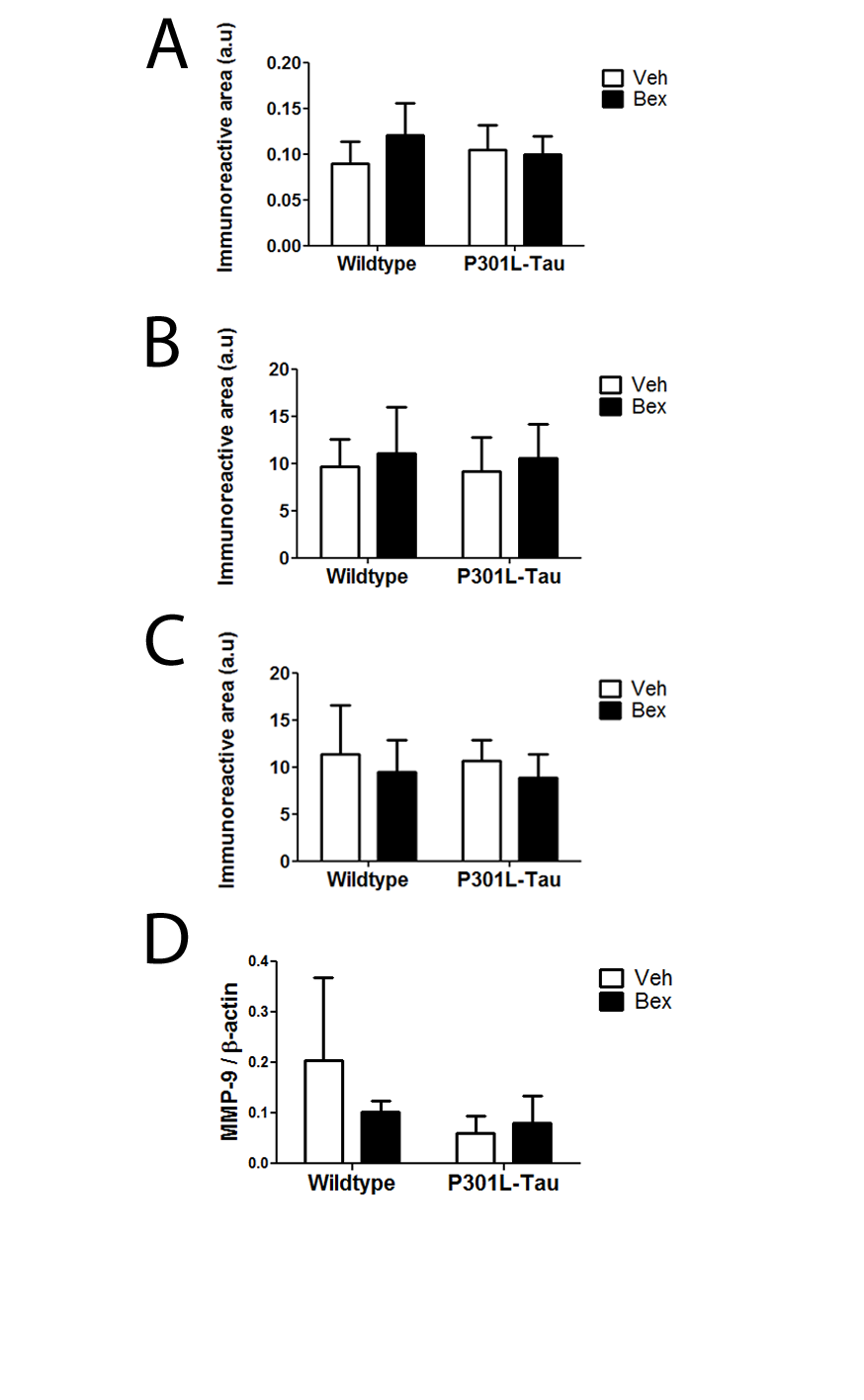


Figure S3


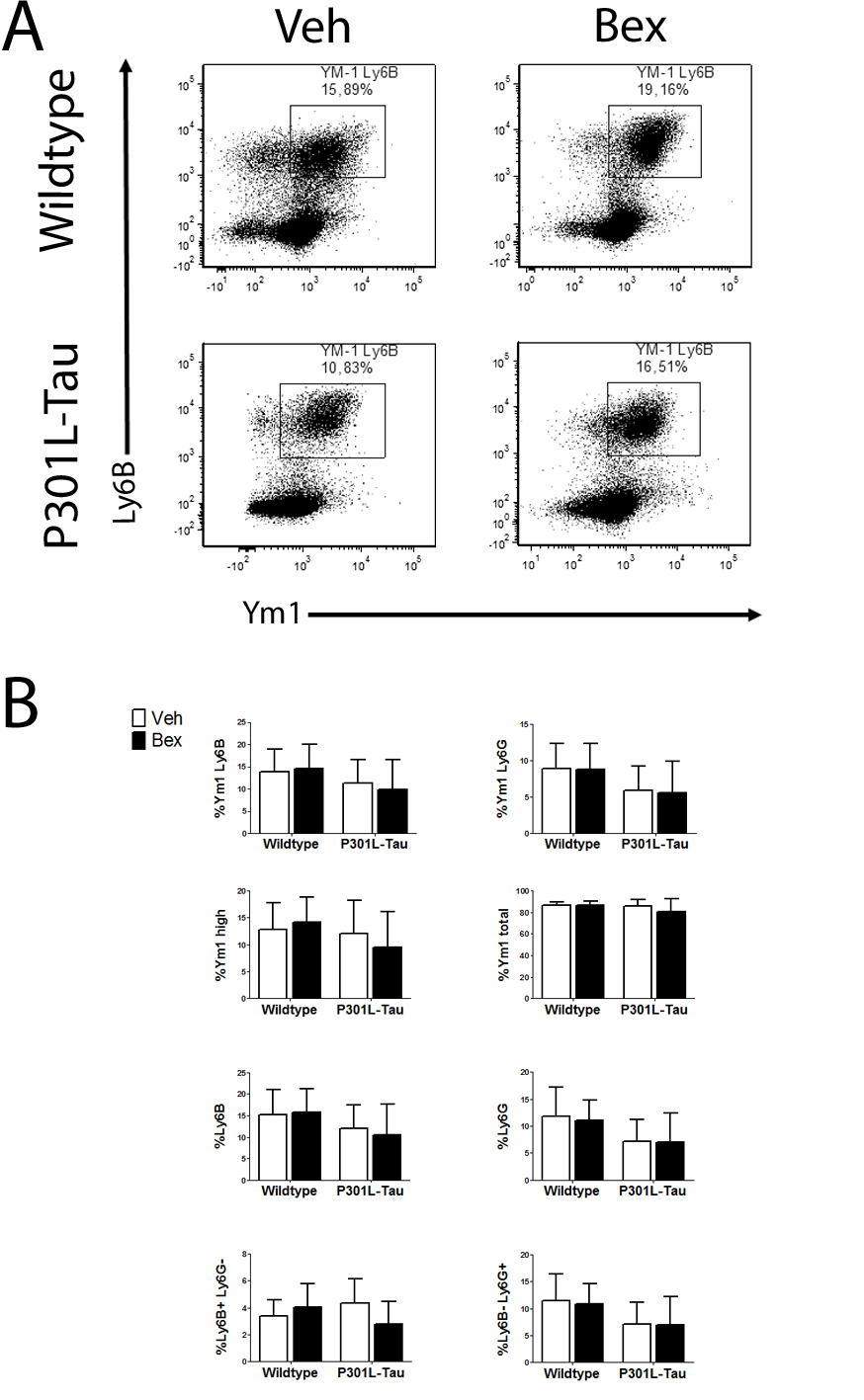


Figure S4


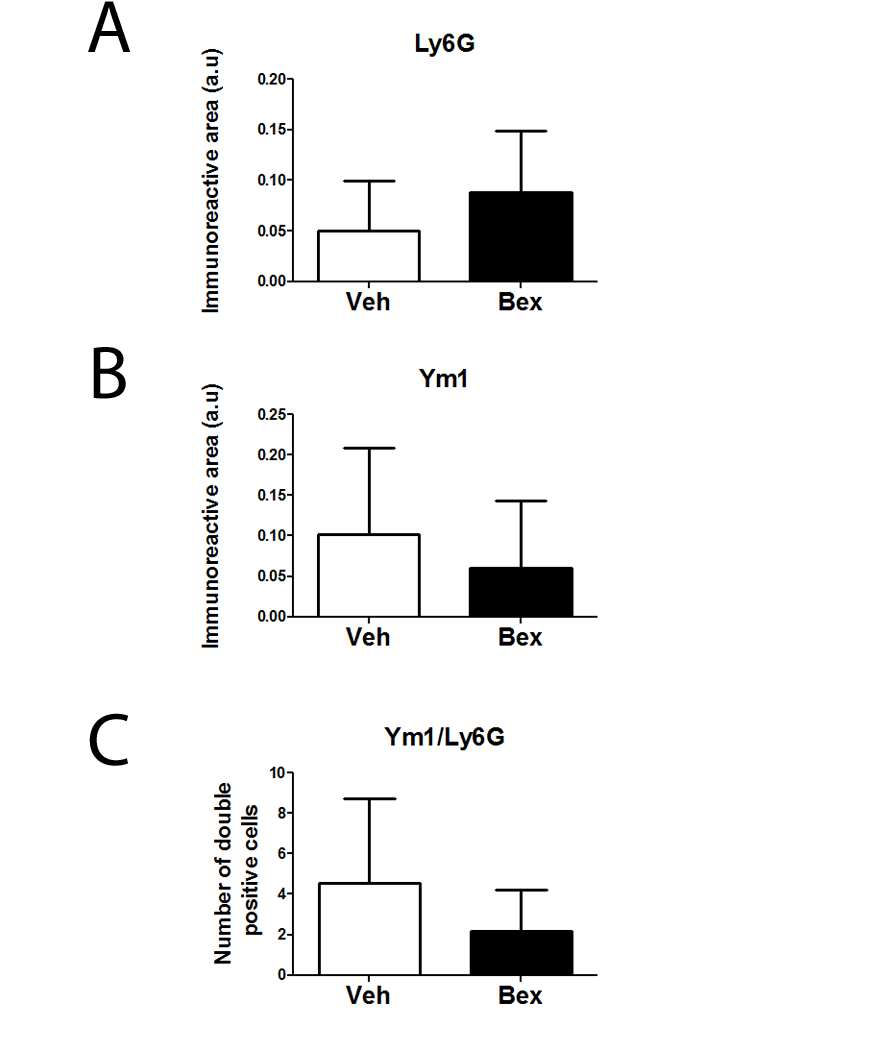


Figure S5


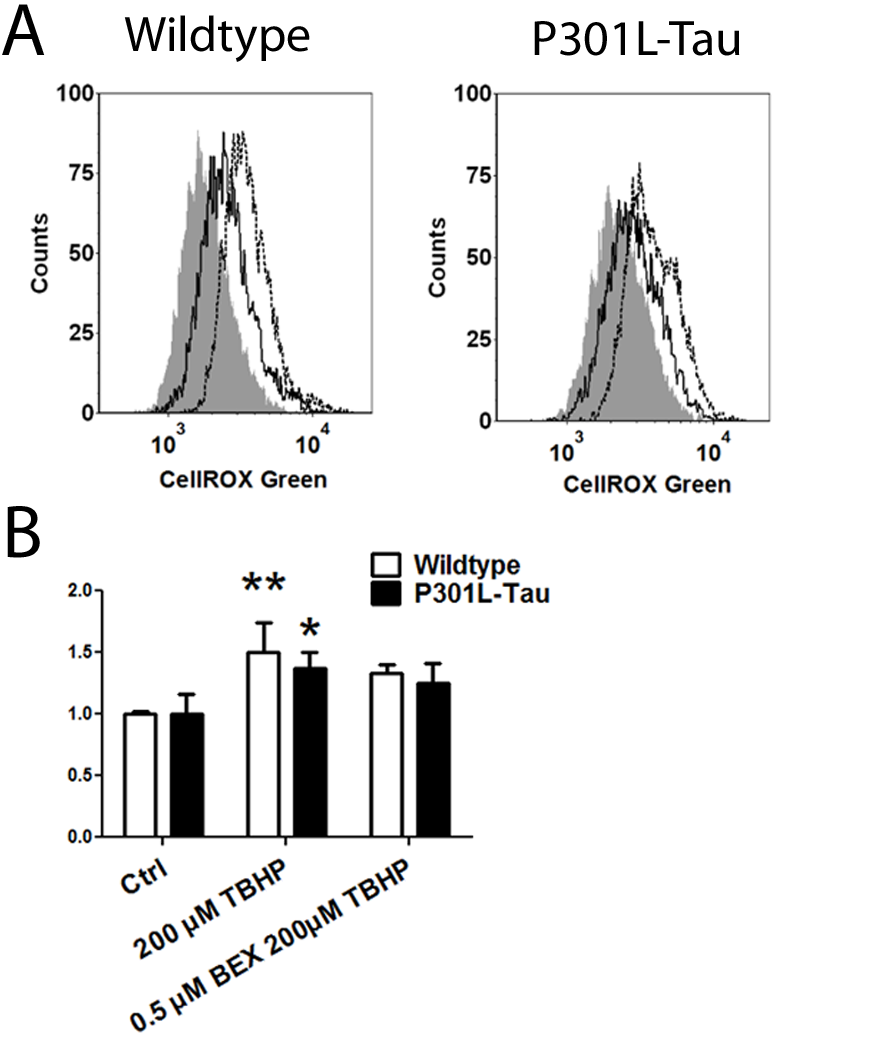

Supplement: Supplementary Information [file srep33176-s1.doc]
